# β-d-Gulose

**DOI:** 10.1107/S1600536814008046

**Published:** 2014-04-16

**Authors:** Tomohiko Ishii, Shunsuke Ohga, Kazuhiro Fukada, Kenji Morimoto, Genta Sakane

**Affiliations:** a Department of Advanced Materials Science, Faculty of Engineering, Kagawa University, 2217-20 Hayashi-cho, Takamatsu, Kagawa 761-0396, Japan; b Department of Applied Biological Science, Faculty of Agriculture, Kagawa University, 2393 Ikenobe, Kagawa 761-0795, Japan; c Department of Chemistry, Faculty of Science, Okayama University of Science, 1-1 Ridaicho, Kita-ku, Okayama 700-0005, Japan

## Abstract

The title compound, C_6_H_12_O_6_, a C-3 position epimer of d-galactose, crystallized from an aqueous solution, was confirmed as β-d-pyran­ose with a ^4^ *C*_1_ (*C1*) conformation. In the crystal, O—H⋯O hydrogen bonds between the hy­droxy groups at the C-1 and C-6 positions connect mol­ecules into a tape structure with an *R*_3_^3^(11) ring motif running along the *a-*axis direction. The tapes are connected by further O—H⋯O hydrogen bonds, forming a three-dimensional network.

## Related literature   

For related structures. see: Fukada *et al.* (2010 ▶). For the chemical synthesis of the title compound, see: Morimoto *et al.* (2013 ▶). For hydrogen-bonding networks, see: Jeffrey & Saenger (1994 ▶); Jeffrey & Mitra (1983 ▶).
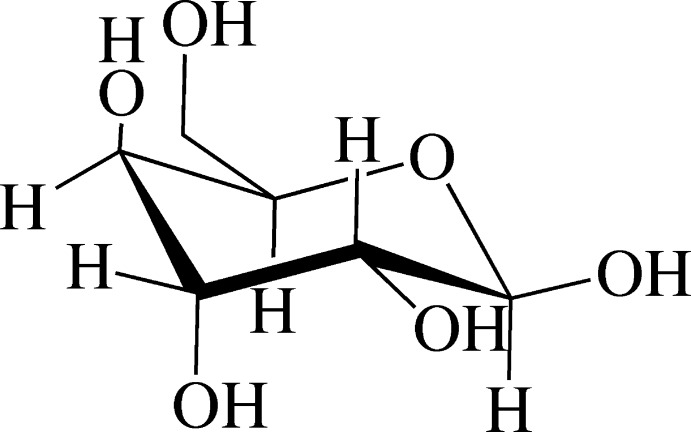


## Experimental   

### 

#### Crystal data  

C_6_H_12_O_6_
*M* *_r_* = 180.16
Orthorhombic, 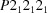

*a* = 7.0800 (3) Å
*b* = 9.8644 (3) Å
*c* = 10.6156 (4) Å
*V* = 741.39 (4) Å^3^
*Z* = 4
Cu *K*α radiation
μ = 1.28 mm^−1^
*T* = 294 K
0.10 × 0.10 × 0.10 mm

#### Data collection  

Rigaku R-AXIS RAPID II diffractometer
Absorption correction: multi-scan (*ABSCOR*; Higashi, 1995 ▶) *T*_min_ = 0.645, *T*_max_ = 0.879
7803 measured reflections
1358 independent reflections
1199 reflections with *F* ^2^ > 2σ(*F* ^2^)
*R*_int_ = 0.070

#### Refinement  

*R*[*F* ^2^ > 2σ(*F* ^2^)] = 0.035
*wR*(*F* ^2^) = 0.073
*S* = 1.05
1358 reflections
116 parameters
H-atom parameters constrained
Δρ_max_ = 0.14 e Å^−3^
Δρ_min_ = −0.14 e Å^−3^

### 

Data collection: *RAPID-AUTO* (Rigaku, 2009 ▶); cell refinement: *RAPID-AUTO*; data reduction: *RAPID-AUTO*; program(s) used to solve structure: *SIR2008* in *Il Milione* (Burla *et al.*, 2007 ▶); program(s) used to refine structure: *SHELXL2013* (Sheldrick, 2008 ▶); molecular graphics: *CrystalStructure* (Rigaku, 2010 ▶); software used to prepare material for publication: *CrystalStructure*.

## Supplementary Material

Crystal structure: contains datablock(s) General, global, I. DOI: 10.1107/S1600536814008046/is5352sup1.cif

Structure factors: contains datablock(s) I. DOI: 10.1107/S1600536814008046/is5352Isup2.hkl

CCDC reference: 903430

Additional supporting information:  crystallographic information; 3D view; checkCIF report

## Figures and Tables

**Table 1:** Hydrogen-bond geometry (Å, °)

*D*—H⋯*A*	*D*—H	H⋯*A*	*D*⋯*A*	*D*—H⋯*A*
O1—H1*A*⋯O6^i^	0.82	1.93	2.736 (3)	168
O2—H2*A*⋯O3^ii^	0.82	2.12	2.785 (3)	139
O3—H3*A*⋯O4^iii^	0.82	1.91	2.722 (3)	173
O4—H4*A*⋯O6^iv^	0.82	2.10	2.915 (3)	173
O6—H6*A*⋯O1^v^	0.82	1.99	2.805 (3)	177

Symmetry codes: (i) 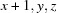; (ii) 
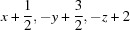
; (iii) 
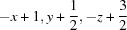
; (iv) 
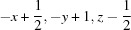
; (v) 
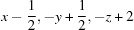
.

**Fig. 1.**
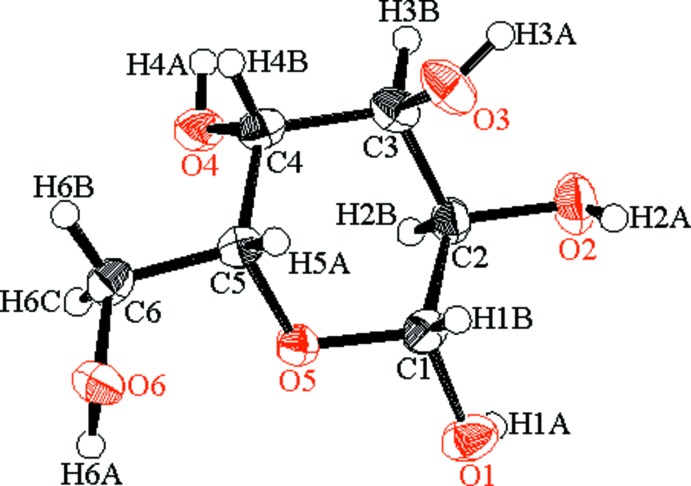
ORTEP view of the title compound with the atom-labeling scheme. The thermal ellipsoids of all non-hydrogen atoms are drawn at the 50% probability level. H atoms are shown as small spheres of arbitrary radius.

**Fig. 2.**
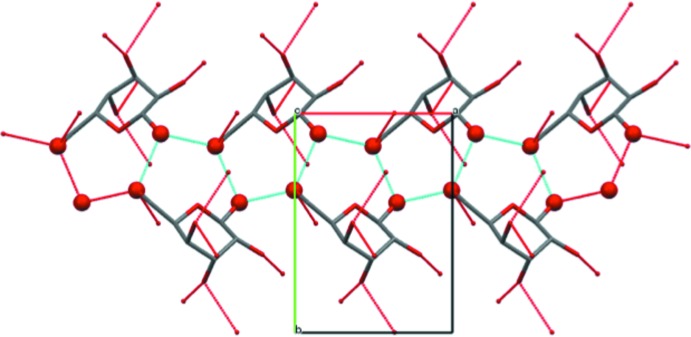
Part of the crystal structure of the title compound with hydrogen-bonding network represented as light blue dashed lines, viewed down the c axis. The hydrogen atoms are omitted for clarity.

## supplementary crystallographic information

### 1. Comment

The crystal system (orthorhombic), space group (*P*2_1_2_1_2_1_), and number of molecules in the unit cell (*Z* = 4) of the title compound are the same as for the typical hexose (C_6_H_12_O_6_) monosaccharides (Fukada *et al.*, 2010). There is a difference in the hydrogen bonding patterns, having a circular chain network returning to the same molecule, and the intermolecular interactions between two adjacent β-D-gulose molecules in the crystal.

In an equatorial OH group at C-2 position, the hydrogen bond can be confirmed as a donor, which connects to the OH group at C-3 position of the neighboring molecule. However, for the axial OH groups at C-3 and C-4 positions, each has hydrogen bonds both as a donor and an acceptor to the OH groups at either the C-2 and C-4, or the C-3 and C-6 positions, respectively. In the OH group at the C-6 position, there is an intermolecular hydrogen bond between the OH group at C-4 position of the neighboring molecule, and there are two additional hydrogen bonds with the OH groups at different C-1 positions in these two different *D*-gulose molecules. There is an infinite hydrogen bonding chain along to the *a*-axis (···O1—H1A···O6—H6A···O1—H1A···), which is connecting to a finite chain (O2—H2A···O3—H3A···O4—H4A···O6—H6A). Therefore, the hydrogen bonding network can be categorized as Jeffrey's class (iv) (Jeffrey & Saenger, 1994; Jeffrey & Mitra, 1983). There is a step for returning to the same gulose molecule in an infinite chain (···gulose O1—H1A···O6—H6A···O1—H1A···gulose O6—H6A···). Such a significant circular hydrogen bonding ring should be treated differently from the typical infinite chain.

### 2. Experimental

*D*-Gulose was prepared from disaccharide lactitol by a combination of microbial and chemical reactions. 3-Ketolactitol, oxidized from lactitol by *Agrobacterium tumefaciens*, was reduced by chemical hydrogenation. The resulting product, *D*-gulosyl-(β-1, 4)-*D*-sorbitol containing *D*-gulose, was hydrolyzed by acid hydrolysis, and its subsequent hydrolysates were separated by chromatography. Lastly, a crude crystal from the concentrated *D*-gulose syrup was recovered by ethanol precipitation, and then its aqueous solution was recrystallized, resulting in pure *D*-gulose. The *D*-gulose was concentrated to a brix value in a range of approximately 85–90%. Ethanol (twice the volume of the resulting syrup) was added and the resulting solution was mixed vigorously. The resulting crystals were dissolved in ultrapure water and then concentrated and crystallized at room temperature. The specific optical rotation of *D*-gulose was analyzed using a polarimeter (JASCO P-1030 Tokyo). An optical rotation was also performed, providing [α]_20_^D^ = -24.10 (authentic sample = -24.74). The ^13^C-NMR spectra of the isolated D-gulose was measured at 600 MHz in D_2_O using an ALPHA 600 system (Jeol Datum, Tokyo). All spectra were collected at 30 °C using trimethylsilyl propanoic acid as internal reference. All of the chemical shifts [δ = 94.6 (C1), 74.5 (C5), 71.9 (C3), 70.2 (C4), 69.8 (C2), 61.7 (C6)] corresponded well with an authentic *D*-gulose sample. These results indicate that the isolated material was *D*-gulose and that the current study was successful in preparing *D*-gulose. The gulose is a specialized member of the rare sugar family, therefore, the details regarding the synthesis, purification, and crystallization of gulose should be reported in a specialized journal (Morimoto *et al.*, 2013).

### 3. Refinement

H atoms bounded to methine-type C (H1B, H2B, H3B, H4B, H5A) were positioned geometrically and refined using a riding model with C—H = 0.98 Å and *U*_iso_(H) = 1.2*U*_eq_(C). H atoms bounded to methylene-type C (H6B, H6C) were positioned geometrically and refined using a riding model with C—H = 0.97 Å and *U*_iso_(H) = 1.2*U*_eq_(C). H atoms bounded to O (H1A, H2A, H3A, H4A, H6A) were positioned geometrically and refined using a riding model with O—H = 0.82 Å and *U*_iso_(H) = 1.2*U*_eq_(O), allowing for free rotation of the OH groups.

### Crystal data

**Table:**

C_6_H_12_O_6_	*F*(000) = 384.00
*M**_r_* = 180.16	*D*_x_ = 1.614 Mg m^−^^3^
Orthorhombic, *P*2_1_2_1_2_1_	Cu *K*α radiation, λ = 1.54187 Å
Hall symbol: P 2ac 2ab	Cell parameters from 7124 reflections
*a* = 7.0800 (3) Å	θ = 4.2–68.2°
*b* = 9.8644 (3) Å	µ = 1.28 mm^−^^1^
*c* = 10.6156 (4) Å	*T* = 294 K
*V* = 741.39 (4) Å^3^	Block, colorless
*Z* = 4	0.10 × 0.10 × 0.10 mm

### Data collection

**Table:**

Rigaku R-AXIS RAPID II diffractometer	1199 reflections with *F*^2^ > 2σ(*F*^2^)
Detector resolution: 10.000 pixels mm^-1^	*R*_int_ = 0.070
ω scans	θ_max_ = 68.2°
Absorption correction: multi-scan (*ABSCOR*; Higashi, 1995)	*h* = −8→8
*T*_min_ = 0.645, *T*_max_ = 0.879	*k* = −11→11
7803 measured reflections	*l* = −12→12
1358 independent reflections	

### Refinement

**Table:**

Refinement on *F*^2^	Hydrogen site location: inferred from neighbouring sites
*R*[*F*^2^ > 2σ(*F*^2^)] = 0.035	H-atom parameters constrained
*wR*(*F*^2^) = 0.073	*w* = 1/[σ^2^(*F*_o_^2^) + (0.0261*P*)^2^] where *P* = (*F*_o_^2^ + 2*F*_c_^2^)/3
*S* = 1.05	(Δ/σ)_max_ < 0.001
1358 reflections	Δρ_max_ = 0.14 e Å^−^^3^
116 parameters	Δρ_min_ = −0.14 e Å^−^^3^
0 restraints	Extinction correction: *SHELXL2013* (Sheldrick, 2008)
Primary atom site location: structure-invariant direct methods	Extinction coefficient: 0.0063 (12)
Secondary atom site location: difference Fourier map	

### Special details

**Table:**

Refinement. Refinement was performed using all reflections. The weighted *R*-factor (*wR*) and goodness of fit (*S*) are based on *F*^2^. *R*-factor (gt) are based on *F*. The threshold expression of *F*^2^ > 2.0 σ(*F*^2^) is used only for calculating *R*-factor (gt).

### Fractional atomic coordinates and isotropic or equivalent isotropic displacement parameters (Å^2^)

**Table:**

	*x*	*y*	*z*	*U*_iso_*/*U*_eq_	
O1	0.6475 (3)	0.4070 (3)	1.02633 (16)	0.0367 (6)	
O2	0.7927 (3)	0.6435 (3)	0.89378 (15)	0.0385 (6)	
O3	0.4219 (3)	0.7683 (2)	0.87238 (18)	0.0369 (6)	
O4	0.3674 (3)	0.49846 (18)	0.64349 (14)	0.0295 (5)	
O5	0.3828 (2)	0.41908 (19)	0.90855 (15)	0.0259 (5)	
O6	−0.0053 (3)	0.35032 (19)	0.92624 (16)	0.0304 (5)	
C1	0.5316 (4)	0.4977 (3)	0.9615 (2)	0.0270 (7)	
C2	0.6282 (4)	0.5724 (3)	0.8553 (2)	0.0257 (6)	
C3	0.4871 (4)	0.6654 (3)	0.7890 (2)	0.0266 (7)	
C4	0.3165 (4)	0.5860 (3)	0.7452 (2)	0.0255 (7)	
C5	0.2385 (4)	0.5021 (3)	0.8521 (2)	0.0234 (6)	
C6	0.0815 (4)	0.4071 (3)	0.8155 (3)	0.0277 (7)	
H1A	0.7468	0.3971	0.9876	0.0441*	
H1B	0.4786	0.5635	1.0210	0.0324*	
H2A	0.7726	0.6811	0.9614	0.0462*	
H2B	0.6682	0.5042	0.7937	0.0308*	
H3A	0.4827	0.8379	0.8608	0.0443*	
H3B	0.5483	0.7077	0.7161	0.0319*	
H4A	0.3971	0.5441	0.5821	0.0354*	
H4B	0.2191	0.6495	0.7164	0.0306*	
H5A	0.1902	0.5641	0.9167	0.0281*	
H6A	0.0367	0.2740	0.9386	0.0364*	
H6B	−0.0125	0.4559	0.7669	0.0333*	
H6C	0.1315	0.3348	0.7634	0.0333*	

### Atomic displacement parameters (Å^2^)

**Table:**

	*U*^11^	*U*^22^	*U*^33^	*U*^12^	*U*^13^	*U*^23^
O1	0.0251 (10)	0.0477 (13)	0.0374 (11)	0.0010 (11)	0.0001 (9)	0.0157 (11)
O2	0.0320 (11)	0.0493 (15)	0.0341 (11)	−0.0151 (10)	−0.0014 (9)	−0.0041 (11)
O3	0.0461 (13)	0.0227 (12)	0.0420 (11)	−0.0050 (10)	0.0113 (10)	−0.0076 (10)
O4	0.0416 (12)	0.0273 (12)	0.0196 (9)	−0.0012 (10)	0.0020 (9)	0.0013 (8)
O5	0.0241 (9)	0.0231 (10)	0.0305 (10)	−0.0011 (9)	−0.0031 (8)	0.0033 (9)
O6	0.0276 (10)	0.0251 (11)	0.0384 (10)	−0.0001 (9)	0.0042 (9)	0.0057 (9)
C1	0.0263 (14)	0.0289 (17)	0.0258 (13)	0.0003 (13)	−0.0030 (13)	0.0012 (12)
C2	0.0230 (13)	0.0283 (16)	0.0257 (13)	−0.0054 (13)	−0.0010 (12)	−0.0013 (13)
C3	0.0302 (15)	0.0242 (17)	0.0253 (13)	−0.0006 (13)	0.0060 (13)	−0.0002 (12)
C4	0.0284 (14)	0.0251 (15)	0.0231 (13)	0.0058 (14)	−0.0004 (12)	0.0016 (13)
C5	0.0225 (13)	0.0247 (15)	0.0231 (12)	0.0041 (11)	0.0019 (12)	0.0011 (12)
C6	0.0265 (14)	0.0320 (17)	0.0248 (13)	0.0019 (14)	0.0006 (11)	0.0001 (13)

### Geometric parameters (Å, º)

**Table:**

O1—C1	1.396 (4)	O1—H1A	0.820
O2—C2	1.420 (3)	O2—H2A	0.820
O3—C3	1.424 (4)	O3—H3A	0.820
O4—C4	1.429 (3)	O4—H4A	0.820
O5—C1	1.424 (3)	O6—H6A	0.820
O5—C5	1.440 (3)	C1—H1B	0.980
O6—C6	1.439 (3)	C2—H2B	0.980
C1—C2	1.510 (4)	C3—H3B	0.980
C2—C3	1.527 (4)	C4—H4B	0.980
C3—C4	1.513 (4)	C5—H5A	0.980
C4—C5	1.510 (4)	C6—H6B	0.970
C5—C6	1.505 (4)	C6—H6C	0.970
			
O3···H2A	2.7927	O6···H4A^viii^	2.0992
O4···H5A	3.2256	H1A···O6^ii^	1.9284
O1···H6A^i^	1.9853	H2A···O3^ix^	2.1169
O2···H6B^ii^	2.6723	H3A···O4^iii^	1.9072
O2···H6C^iii^	2.5757	H3B···O5^iii^	2.5179
O3···H2A^iv^	2.1169	H4A···O6^vi^	2.0992
O4···H3A^v^	1.9072	H5A···O4^viii^	2.5185
O4···H5A^vi^	2.5185	H6A···O1^x^	1.9853
O5···H3B^v^	2.5179	H6B···O2^vii^	2.6723
O6···H1A^vii^	1.9284	H6C···O2^v^	2.5757
			
C1—O5—C5	112.3 (2)	C6—O6—H6A	109.480
O1—C1—O5	106.3 (2)	O1—C1—H1B	109.359
O1—C1—C2	114.5 (2)	O5—C1—H1B	109.362
O5—C1—C2	107.82 (18)	C2—C1—H1B	109.363
O2—C2—C1	113.41 (19)	O2—C2—H2B	107.098
O2—C2—C3	111.9 (3)	C1—C2—H2B	107.103
C1—C2—C3	109.9 (2)	C3—C2—H2B	107.097
O3—C3—C2	110.75 (19)	O3—C3—H3B	109.292
O3—C3—C4	107.5 (2)	C2—C3—H3B	109.290
C2—C3—C4	110.7 (3)	C4—C3—H3B	109.286
O4—C4—C3	110.1 (2)	O4—C4—H4B	109.124
O4—C4—C5	109.2 (3)	C3—C4—H4B	109.127
C3—C4—C5	110.15 (19)	C5—C4—H4B	109.123
O5—C5—C4	111.4 (2)	O5—C5—H5A	108.140
O5—C5—C6	106.1 (3)	C4—C5—H5A	108.132
C4—C5—C6	114.7 (2)	C6—C5—H5A	108.139
O6—C6—C5	110.29 (19)	O6—C6—H6B	109.601
C1—O1—H1A	109.471	O6—C6—H6C	109.595
C2—O2—H2A	109.468	C5—C6—H6B	109.603
C3—O3—H3A	109.466	C5—C6—H6C	109.595
C4—O4—H4A	109.475	H6B—C6—H6C	108.127
			
C1—O5—C5—C4	61.5 (3)	C1—C2—C3—C4	−55.0 (3)
C1—O5—C5—C6	−173.06 (15)	O3—C3—C4—O4	168.99 (18)
C5—O5—C1—O1	172.54 (16)	O3—C3—C4—C5	−70.5 (3)
C5—O5—C1—C2	−64.2 (3)	C2—C3—C4—O4	−69.9 (3)
O1—C1—C2—O2	−55.5 (3)	C2—C3—C4—C5	50.5 (3)
O1—C1—C2—C3	178.41 (18)	O4—C4—C5—O5	68.1 (3)
O5—C1—C2—O2	−173.59 (19)	O4—C4—C5—C6	−52.5 (3)
O5—C1—C2—C3	60.4 (3)	C3—C4—C5—O5	−53.0 (3)
O2—C2—C3—O3	−62.8 (3)	C3—C4—C5—C6	−173.52 (19)
O2—C2—C3—C4	178.08 (16)	O5—C5—C6—O6	66.8 (3)
C1—C2—C3—O3	64.1 (3)	C4—C5—C6—O6	−169.7 (2)

Symmetry codes: (i) *x*+1/2, −*y*+1/2, −*z*+2; (ii) *x*+1, *y*, *z*; (iii) −*x*+1, *y*+1/2, −*z*+3/2; (iv) *x*−1/2, −*y*+3/2, −*z*+2; (v) −*x*+1, *y*−1/2, −*z*+3/2; (vi) −*x*+1/2, −*y*+1, *z*−1/2; (vii) *x*−1, *y*, *z*; (viii) −*x*+1/2, −*y*+1, *z*+1/2; (ix) *x*+1/2, −*y*+3/2, −*z*+2; (x) *x*−1/2, −*y*+1/2, −*z*+2.

### Hydrogen-bond geometry (Å, º)

**Table:**

*D*—H···*A*	*D*—H	H···*A*	*D*···*A*	*D*—H···*A*
O1—H1*A*···O6^ii^	0.82	1.93	2.736 (3)	168
O2—H2*A*···O3^ix^	0.82	2.12	2.785 (3)	139
O3—H3*A*···O4^iii^	0.82	1.91	2.722 (3)	173
O4—H4*A*···O6^vi^	0.82	2.10	2.915 (3)	173
O6—H6*A*···O1^x^	0.82	1.99	2.805 (3)	177

Symmetry codes: (ii) *x*+1, *y*, *z*; (iii) −*x*+1, *y*+1/2, −*z*+3/2; (vi) −*x*+1/2, −*y*+1, *z*−1/2; (ix) *x*+1/2, −*y*+3/2, −*z*+2; (x) *x*−1/2, −*y*+1/2, −*z*+2.
